# VaxiJen: a server for prediction of protective antigens, tumour antigens and subunit vaccines

**DOI:** 10.1186/1471-2105-8-4

**Published:** 2007-01-05

**Authors:** Irini A Doytchinova, Darren R Flower

**Affiliations:** 1 Faculty of Pharmacy, Medical University of Sofia, 2 Dunav St., 1000 Sofia, Bulgaria; 2 The Jenner Institute, Oxford University, Compton, Berkshire, RG20 7NN, UK

## Abstract

**Background:**

Vaccine development in the post-genomic era often begins with the *in silico *screening of genome information, with the most probable protective antigens being predicted rather than requiring causative microorganisms to be grown. Despite the obvious advantages of this approach – such as speed and cost efficiency – its success remains dependent on the accuracy of antigen prediction. Most approaches use sequence alignment to identify antigens. This is problematic for several reasons. Some proteins lack obvious sequence similarity, although they may share similar structures and biological properties. The antigenicity of a sequence may be encoded in a subtle and recondite manner not amendable to direct identification by sequence alignment. The discovery of truly novel antigens will be frustrated by their lack of similarity to antigens of known provenance. To overcome the limitations of alignment-dependent methods, we propose a new alignment-free approach for antigen prediction, which is based on auto cross covariance (ACC) transformation of protein sequences into uniform vectors of principal amino acid properties.

**Results:**

Bacterial, viral and tumour protein datasets were used to derive models for prediction of whole protein antigenicity. Every set consisted of 100 known antigens and 100 non-antigens. The derived models were tested by internal leave-one-out cross-validation and external validation using test sets. An additional five training sets for each class of antigens were used to test the stability of the discrimination between antigens and non-antigens. The models performed well in both validations showing prediction accuracy of 70% to 89%. The models were implemented in a server, which we call VaxiJen.

**Conclusion:**

VaxiJen is the first server for alignment-independent prediction of protective antigens. It was developed to allow antigen classification solely based on the physicochemical properties of proteins without recourse to sequence alignment. The server can be used on its own or in combination with alignment-based prediction methods. It is freely-available online at the URL: .

## Background

Vaccination is a highly effective approach to disease control in human and veterinary health care. A vaccine is a molecular or supramolecular agent which elicits specific, protective immunity; that is an enhanced adaptive immune response to re-infection by pathogenic microbes through the potentiation of immune memory. Vaccination ultimately mitigates the effect of subsequent infection and disease. Thus, the immune system recognizes vaccine agents as foreign, destroys them, and subsequently 'remembers' them. When the pathogenic microorganism is encountered again, the immune system has been primed to respond, by neutralizing the target before it can enter cells, or/and by destroying infected cells before the microorganism can grow and cause damage. Vaccines have contributed to the eradication of smallpox, the near eradication of polio, and the control of a variety of diseases, including rubella, measles, mumps, chickenpox, typhoid [1].

Vaccines from the pre-genomic era were based on killed or live, but attenuated, microorganisms, or subunits purified from them [2]. Subunit vaccines contain one or more pure or semi-pure antigens. In order to develop subunit vaccines, it is critical to identify those proteins which are important for inducing protection and to eliminate others. An antigen is said to be protective if it is able to induce protection from subsequent challenge by a disease-causing infective agent in an appropriate animal model following immunization. The empirical approach to sub-unit vaccine development, which includes several steps, begins with pathogen cultivation, followed by purification into components, and then testing of antigens for protection [3]. Apart from being time- and labour-consuming, this approach has several limitations that can lead to failure. Vaccines can not be developed using this approach for microorganisms which can not easily be cultured and only allows for the identification of those antigens which can be obtained in sufficient quantities. In some cases, the most abundant proteins are not immunoprotective. In other cases, the antigen expressed during *in vivo *infection is not expressed during *in vitro *cultivation.

Genomics has revolutionized vaccine research. The ability to sequence the whole genome of a virulent microorganism has led some to screen *in silico *for the most probable protective antigens before undertaking confirmatory experiments. This approach, known as reverse vaccinology [4], was first used to identify antigens as potential candidate vaccines against serogroup B meningococcus [5]. Apart from obvious advantages – such as speed and low cost – the success of this approach is dependent on the accuracy of antigen prediction, and many bioinformatics tools are available to facilitate this process [6-8]. They can identify surface-associated or outer membrane proteins, signal peptides, lipoproteins, or host-cell binding domains. Most algorithms use sequence alignment to identify antigens. This is problematic for several reasons. Some proteins formed through divergent or convergent evolution lack obvious sequence similarity, although they may share similar structures and biological properties [9]. In such a situation, alignment-based approaches may produce ambiguous results or fail. Moreover, antigenicity, as a property, may be encoded in a sequence in a subtle and recondite manner not amendable to direct identification by sequence alignment. Likewise, the discovery of truly novel antigens will be frustrated by their lack of similarity to antigens of known provenance.

To overcome the limitations of alignment-dependent sequence similarity methods, we propose a new alignment-independent method for antigen prediction based on auto cross covariance (ACC) transformation of protein sequences into uniform equal-length vectors. ACC is an protein sequence mining method developed by Wold et al. [10], which has been applied to quantitative structure-activity relationships (QSAR) studies of peptides with different length [11,12] and for protein classification [13]. The ACC transformation accounts for neighbour effects, i.e. the lack of independence between different sequence positions. In the present study, we applied ACC pre-processing to sets of known bacterial, viral and tumour antigens and developed alignment-independent models for antigen recognition based on the main chemical properties of amino acid sequences. The principal properties of the amino acids were represented by *z *descriptors, originally derived by Hellberg et al. [14] to describe amino acid hydrophobicity, molecular size and polarity. The models were implemented in a server for the prediction of protective antigens and subunit vaccines, which we call VaxiJen. This is freely accessible via the World Wide Web. Our method is the first alignment-free bioinformatics tool for the *in silico *identification of antigens.

## Results

Three datasets were used in this study: one for bacteria, one for viruses, and one for tumours. Each set consisted of 100 known antigens and 100 non-antigens, collected as described in the Methods section. Each amino acid in the protein sequence was represented by three *z *descriptors: *z*_1_, *z*_2_, and *z*_3_. Each protein was transformed into a uniform vector, which consisted of 45 ACC terms, by applying ACC pre-processing, as described in the Methods section. The new matrices were imported into SIMCA-P 8.0 [15] and were subject to a two-class discriminant analysis using the partial least squares technique (DA-PLS). The models were validated using leave-one-out cross-validation (LOO-CV) on the whole sets and by external validation using test sets. The test sets were selected randomly to include 25% of the whole sets. Then models were developed based on the remaining 75% and tested on the excluded proteins. The validation results were assessed in terms of *AUC*_*ROC*_, *accuracy*, *sensitivity *and *specificity*, as described in the Methods section. Additionally, five negative sets were compiled, and subsequently combined with the positive set to generate five new training sets. They also underwent DA-PLS and their *AUC*_*ROC*_, *accuracy*, *sensitivity *and *specificity *are given as mean values. Within the server, the final model for each type was derived as a mean of the best five models, as assessed by LOO-CV.

### VaxiJen model for prediction of protective bacterial antigens

**Figure 1:**
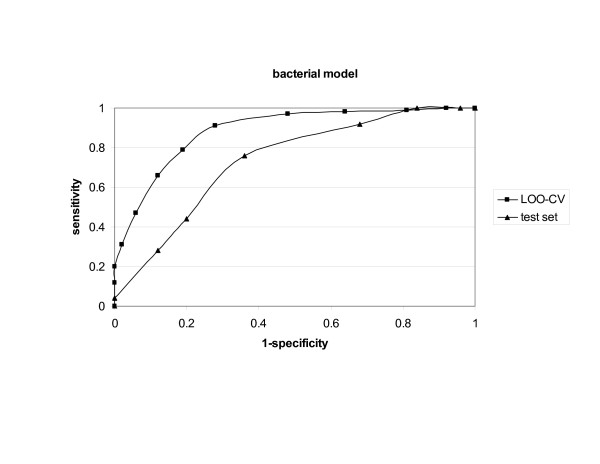
ROC curves for VaxiJen bacterial model.

The LOO-CV of the bacterial model had 82% *accuracy*, 91% *sensitivity *and 72% *specificity *(Table 1). As expected, the external validation showed a lower value but was still satisfactory. The *ROC *curves are shown in Figure 1. The average values for the additional sets were very close to those derived for the initial model.

**Table 1:** VaxiJen models validation.

*model*	*validation*	AUC_ROC_^a^	*threshold*^b^	accuracy%^c^	sensitivity%^d^	specificity%^e^
	LOO-CV	0.883	0.5	80	79	81
bacterial	test set	0.726	0.5	70	76	64
	LOO-CV (mean)^g^	0.899	0.5	83	81	85
	LOO-CV	0.937	0.5	87	91	82
viral	test set	0.743	0.4	70	84	56
	LOO-CV (mean)^g^	0.810	0.5	73	74	71
	LOO-CV	0.964	0.5	89	94	84
tumour	test set	0.930	0.5	86	96	76
	LOO-CV (mean)^g^	0.911	0.5	82	78	86

^a^The area under the curve (*AUC*_*ROC*_) is a quantitative measure of the predictive ability and varies from 0.5 for a random prediction to 1.0 for a perfect prediction.

^b^The threshold of the highest accuracy.

^c^*Accuracy *= (true antigens + true non-antigens)/total.

^d^*Sensitivity *= true antigens/all antigens.

^e^*Specificity *= true non-antigens/all non-antigens.

^g^Mean values of five training sets.

### VaxiJen model for prediction of protective viral antigens

**Figure 2:**
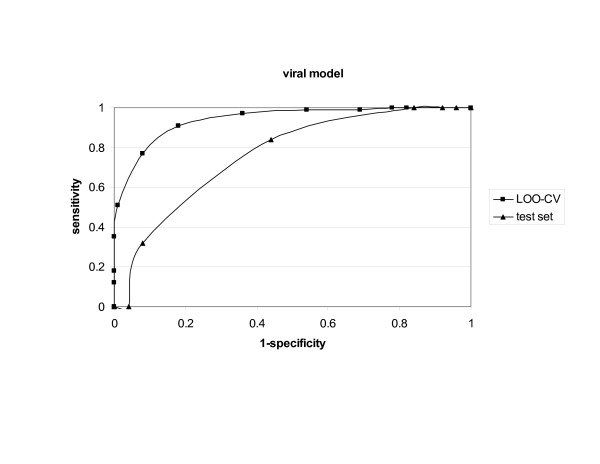
ROC curves for VaxiJen viral model.

The viral model performed very well in the LOO-CV (87% *accuracy*); performance in the external validation was more moderate (70% *accuracy *at threshold 0.4) (Table 1). *ROC *curves of the viral model validation are shown in Figure 2. The additional training sets showed lower mean *accuracy*, *sensitivity *and *specificity*.

### VaxiJen model for prediction of tumour antigens

**Figure 3:**
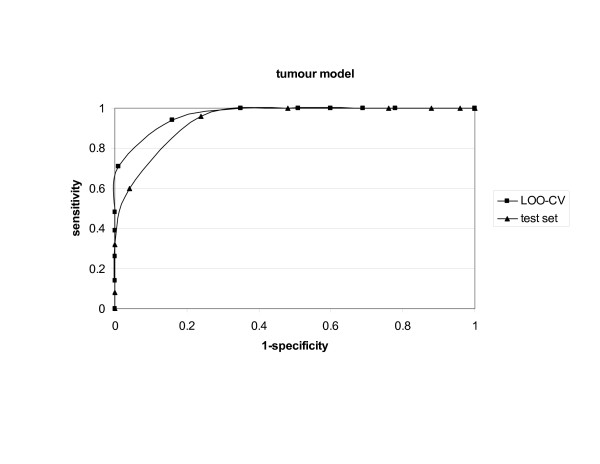
ROC curves for VaxiJen tumour model.

The tumour model had excellent performance both in the LOO-CV and in the external validation, exhibiting more than 85% *accuracy*. The *ROC *curves are shown in Figure 3. The additional models had lower *sensitivity *but similar *specificity *and *accuracy*.

### Sequence similarity of training set

Potential similarity between sequences in the antigen and non-antigen sets was assessed as described. The viral and bacterial protective antigen sequence sets show very little sequence similarity. This reflects their diverse species origins. The tumour set, derived from a single proteome, exhibits a higher internal degree of self-similarity, but is still clearly highly diverse.

### VaxiJen server

The LOO-CV bacterial, viral and tumour models were included in the VaxiJen server. Protein sequences can be submitted as single proteins or uploaded as a multiple sequence file in fasta format. A single target organism can be selected. Additionally, ACC coefficients can be output. This option makes the server useful for general ACC calculations of proteins. The results page lists the selected target, the protein sequence, its prediction probability, and a statement of protective antigen or non-antigen, according to a predefined cutoff. Since more of the models had their highest accuracy at a threshold of 0.5, this threshold value was chosen for all types.

## Discussion

VaxiJen is the first server for alignment-independent prediction of protective antigens of bacterial, viral and tumour origin. The server contains models derived by ACC pre-processing of amino acids properties. The predictive ability of our models was tested by internal leave-one-out cross-validation on training sets and by external validation on test sets. Accuracies of internal and external validation for the three models lie in the range 70% to 89%. The models showed remarkable stability, as tested by combinations of the positive set and five different negative sets. Thus, VaxiJen is a reliable and consistent tool for the prediction of protective antigens. It can be used singly or in combination with other bioinformatics tools used for reverse vaccinology.

The *z *descriptors are highly condensed descriptors, and are derived from a principal component analysis (PCA) of 29 experimental or calculated physicochemical properties of the twenty naturally occurring amino acids. They correspond to the first three principal components explaining the variance in the set [14]: *z*_1 _represents hydrophobicity, *z*_2 _steric properties, and *z*_3 _polarity of the amino acids. Since their creation, *z *descriptors have been widely used for the characterization [16] and classification [13] of proteins, and in QSAR studies on peptides [17,18]. Recently, we have found that *z *descriptors are good predictors of MHC binding peptides [19,20]. In the present study, *z *descriptors represent the main physicochemical properties important for the recognition of antigens.

ACC transformations were used to remove irrelevant information, such as sequence length, and to amplify the class-discriminating properties [10]. Sjostrom et al [16] applied the ACC transformation to *z *scale values in order to assign successfully the subcellular location of bacterial proteins (i.e. cytoplasmic, inner membrane, periplasm, or outer membrane). More recently, a similar method was applied to G-protein coupled receptors (GPCRs) and succeeded in classifying them into their major classes [13]. As antigenicity is not a simple, readily-interpreted linear property, it is unsurprising that ACC pre-processing of the physicochemical properties of antigens and non-antigens allows for a good discrimination between them. The recognition of protective antigens arises synergistically from a combination of intermolecular interactions which involves a diverse variety of underlying features – steric, electrostatic and hydrophobic – which are explained well by the three *z *descriptors.

The most important result of the present work is the ability of the models to predict whether a protein sequence will, or will not, be a protective antigen. Such antigens form the basis of subunit vaccines. In order to facilitate the use of the derived models, a server, named VaxiJen, was developed to allow users to assess a protein's ability to induce protection. The server deals with single proteins as well as whole proteomes submitted in fasta format. As the method is general, models for parasite and fungal antigens will be developed in the future and included in the VaxiJen server.

## Conclusion

VaxiJen is the first server for alignment-independent prediction of protective antigens. It was developed to allow antigen classification based solely on the physicochemical properties of the protein irrespective of sequence length and the need for alignment. VaxiJen is an open system: new models will be included in the future, old ones will be improved. The server can be used singly or in combination with alignment-dependent prediction methods.

## Methods

### Protein datasets

Three datasets were used: one for bacteria, one for viruses and one for tumours. The sets are given as part of Additional Material. Each set consists of 100 known antigens and 100 non-antigens. The bacterial and viral antigens were collected from the literature. A protein was identified as an antigen if it (or part of it) has been shown to induce a protective response in an appropriate animal model after immunization. Tumour antigens were collected from the SEREX database available within the Cancer Immunome Database [21].

The sets of non-antigens were constructed to mirror the antigen sets. The bacterial non-antigen set contained proteins randomly selected from the same set of species. The viral non-antigen set was compiled from viral proteomes downloaded from the Viral Bioinformatics Resource Center [22]. Because, on average, viral genomes are so small, a variant method was used to select non-antigens. Proteins were selected at random, but care was taken that sequences were not obviously related at the sequence level to members of the positive set or to each other. A BLAST expectation value of 3.0 was used: sequences were only accepted which had a value more positive than this cutoff. As each new sequence was assessed, it was compared to both the positive set of known antigens and the growing list of non-antigens. The tumour non-antigen set included randomly chosen human proteins. Proteomes and protein sequences were obtained from the UniProt Knowledgebase of the ExPASy Proteomics Server [23]. For the external validation of the three models, test sets of 25 antigens and 25 non-antigens were selected by picking every fourth protein in the database sorted alphabetically according to the protein swiss-prot number, vprcpep ID, or SEREX ID. To test the stability of the models, five additional negative sets for each kingdom were compiled algorithmically. These sets were combined with the corresponding positive set to generate five new training sets. These sets underwent the same DA-PLS and the derived models were compared with the initial one in terms of *AUC*_*ROC*_, *accuracy*, *sensitivity *and *specificity*. The three positive sets are available as supplementary material [see Additional file 1].

### z descriptors

The *z *descriptors, defined by Hellberg and collaborator [14], summarize the principal physicochemical properties of the amino acids. These descriptors were derived by principal component analysis of a data matrix consisting of 29 molecular descriptors, like molecular weight, pK_a_s, ^13^C NMR shifts, etc. The first principle component (*z*_1_) reflects the hydrophobicity of amino acids, the second (*z*_2_) their size, and the third (*z*_3_) their polarity. By arranging the *z *values according to the amino acid sequence, it is possible to quantify the structural variations numerically within a series of related proteins. In the present study the *z*_1_, *z*_2 _and *z*_3 _descriptors were used to describe the protein sequences.

### Auto cross covariance (ACC) pre-processing

As the proteins used in the study had different lengths, an auto cross covariance (ACC) transformation was used to transform them to a uniform length. The auto covariance Ajj(lag) was calculated according to Eqn. (1) [10]:

Ajj(l)=∑in−lzj,i×zj,i+1n−l     Eqn. (1)
 MathType@MTEF@5@5@+=feaafiart1ev1aaatCvAUfKttLearuWrP9MDH5MBPbIqV92AaeXatLxBI9gBaebbnrfifHhDYfgasaacH8akY=wiFfYdH8Gipec8Eeeu0xXdbba9frFj0=OqFfea0dXdd9vqai=hGuQ8kuc9pgc9s8qqaq=dirpe0xb9q8qiLsFr0=vr0=vr0dc8meaabaqaciaacaGaaeqabaqabeGadaaakeaacqWGbbqqcqWGQbGAcqWGQbGAcqGGOaakcqWGSbaBcqGGPaqkcqGH9aqpdaaeWbqaamaalaaabaGaemOEaO3aaSbaaSqaaiabdQgaQjabcYcaSiabdMgaPbqabaGccqGHxdaTcqWG6bGEdaWgaaWcbaGaemOAaOMaeiilaWIaemyAaKMaey4kaSIaeGymaedabeaaaOqaaiabd6gaUjabgkHiTiabdYgaSbaaaSqaaiabdMgaPbqaaiabd6gaUjabgkHiTiabdYgaSbqdcqGHris5aOGaaCzcaiaaxMaacqqGfbqrcqqGXbqCcqqGUbGBcqGGUaGlcqqGGaaidaqadaqaaiabigdaXaGaayjkaiaawMcaaaaa@576B@

Index *j *was used for the *z*-scales (*j *= 1, 2, 3), *n *is the number of amino acids in a sequence, index *i *is the amino acid position (*i *= 1, 2, ...n) and *l *is the lag (*l *= 1, 2, ...*L*). In order to investigate the influence of close amino acid proximity on protein antigenicity, a short range of lags (*L *= 1, 2, 3, 4, 5) were used. Cross covariances – Cjk(lag) – between two different *z*-scales, *j *and *k*, were calculated according to Eqn. (2) [10]:

Cjk(l)=∑in−lzj,i×zk,i+1n−l     Eqn. (2)
 MathType@MTEF@5@5@+=feaafiart1ev1aaatCvAUfKttLearuWrP9MDH5MBPbIqV92AaeXatLxBI9gBaebbnrfifHhDYfgasaacH8akY=wiFfYdH8Gipec8Eeeu0xXdbba9frFj0=OqFfea0dXdd9vqai=hGuQ8kuc9pgc9s8qqaq=dirpe0xb9q8qiLsFr0=vr0=vr0dc8meaabaqaciaacaGaaeqabaqabeGadaaakeaacqWGdbWqcqWGQbGAcqWGRbWAcqGGOaakcqWGSbaBcqGGPaqkcqGH9aqpdaaeWbqaamaalaaabaGaemOEaO3aaSbaaSqaaiabdQgaQjabcYcaSiabdMgaPbqabaGccqGHxdaTcqWG6bGEdaWgaaWcbaGaem4AaSMaeiilaWIaemyAaKMaey4kaSIaeGymaedabeaaaOqaaiabd6gaUjabgkHiTiabdYgaSbaaaSqaaiabdMgaPbqaaiabd6gaUjabgkHiTiabdYgaSbqdcqGHris5aOGaaCzcaiaaxMaacqqGfbqrcqqGXbqCcqqGUbGBcqGGUaGlcqqGGaaidaqadaqaaiabikdaYaGaayjkaiaawMcaaaaa@5775@

The results of these transformations were new uniform sets of 45 variables (3^2 ^× 5) for each protein.

### Discriminant analysis by partial least squares (DA-PLS)

Two-class discriminant analysis by partial least squares (DA-PLS), as implemented in SIMCA-P 8.0 [17], was applied to the matrices, which consisted of 45 variables and 200 observations (100 antigens + 100 non-antigens). The optimum number of components was selected by adding components until the next component to be added explained less than 10% of the variance. The predictive accuracy of the models was measured by leave-one-out cross-validation (LOO-CV) on the whole set and by external validation on the test set using Receiver Operating Characteristic (*ROC*) curves [26]. The correctly predicted antigens and non-antigens were defined as true positives (TP) and true negatives (TN), respectively, while the incorrectly predicted antigens and non-antigens yielded false negatives (FN) and false positives (FP), respectively. Two variables – *sensitivity *[TP/(TP + FN)] and *1-specificity *[FP/(TN + FP)] – were calculated at different thresholds and *ROC *curves were generated [24]. The area under the curve (*AUC*_*ROC*_) is a quantitative measure of the predictive ability and varies from 0.5 for a random prediction to 1.0 for a perfect prediction. Prediction *accuracy *[(TP + TN)/total] at different thresholds was also calculated.

### Sequence similarity of training set

Potential similarity between sequences in the antigen and non-antigen sets could bias the LOO-CV. Using a standard cutoff [25], all sequences from the positive set were compared against all other positive sequences using BLAST [6]. Using lists of hits to define nearest-neighbour connections, the algorithm of Floyd [26] was used to cluster the sequences. The results are shown in Table 2.

**Table 2:** Similarities between sequences in the three training sets.

**Model Type**	**Number of clusters**	**Minimum cluster size**	**Maximum cluster size**	**Number of Singletons**	**Average cluster size**	**Cluster distribution**^a^
Bacterial	84	1	4	74	1.19	6,2,2
Viral	87	1	5	78	1.15	7,1,0,1
Tumour	76	1	7	66	1.32	4,2,2,1,0,1

For a given cut-off, a perfectly diverse set of sequences will have number of clusters equal to the number of sequences, a maximum and minimum cluster size of one, and an average cluster size of one.

^a ^for non-singleton clusters of 2 or more members. Cluster numbers are shown in ascending cluster size.

### VaxiJen server

The VaxiJen server [27] is implemented in Perl, with an interface written in HTML. VaxiJen identifies bacterial, viral and tumour antigens using three different models, derived in the present study. Protein sequences are uploaded as single or multiple files in plain or fasta format respectively. The results page reports antigen probability (as a fraction of unity) for each protein and a statement of antigen status ("probable Antigen" versus "Probable Non-Antigen").

## Availability and requirements

Project name: VaxiJen

Project home page: 

Operating system(s): IRIX, Linux, Windows

Programming language: Perl

Other requirements: none

License: free

Any restrictions to use by non-academics: none

## Authors' contributions

IAD derived and tested the models included in this study. DRF designed and implemented the web server. Both authors were involved in compilation of data sets. Both authors have read and approved the final manuscript.

## Supplementary Material

Additional File 1 containing the bacterial, viral and tumour sets of antigen and non-antigens used in the study. Each protein in the datasets is given with its origin species, name, ID number (swiss-prot, vbrc or SEREX) and reference. Click here for file
